# The role of PANoptosis in diabetes and its complications: mechanisms and therapeutic prospects

**DOI:** 10.3389/fimmu.2026.1902219

**Published:** 2026-08-19

**Authors:** Jinhong Yan, Qiuyue Wang, Fenqin Chen

**Affiliations:** 1 Department of Geriatrics, The First Hospital of China Medical University, Shenyang, China; 2 Department of Endocrinology, The First Hospital of China Medical University, Shenyang, China

**Keywords:** diabetes mellitus, diabetic complications, PANoptosis, PANoptosome, programmed cell death, therapeutic strategies

## Abstract

Diabetes mellitus and its complications are chronic inflammatory diseases driven by metabolic stress. PANoptosis is a recently defined inflammatory lytic cell death pathway that integrates key features of pyroptosis, apoptosis, and necroptosis, and is orchestrated by the PANoptosome complex. Emerging evidence indicates that PANoptosis plays a critical role in the pathogenesis of diabetic complications, prominently in diabetic kidney disease, retinopathy, neuropathy, and cardiomyopathy, with emerging indirect evidence from surrogate models suggesting its potential involvement in diabetic foot ulcers. In this review, we summarise the core molecular mechanisms of PANoptosis, with a focus on the crosstalk among the three programmed cell death pathways under diabetic conditions. We discuss how metabolic stressors such as hyperglycaemia, lipotoxicity, and endoplasmic reticulum stress activate distinct PANoptosome assemblies—including those involving Z-DNA-binding protein 1 (ZBP1), absent in melanoma 2 (AIM2), receptor-interacting protein kinase 1 (RIPK1), NOD-like receptor family pyrin domain-containing protein 12 (NLRP12), NOD-like receptor family pyrin domain-containing protein 3 (NLRP3), and NOD-like receptor family CARD domain-containing protein 5 (NLRC5)—thereby linking metabolic dysregulation to inflammatory cell death. Moreover, we highlight recent advances in targeting PANoptosis as a therapeutic strategy, emphasising interventions directed at upstream metabolic triggers, PANoptosome components, and downstream effector molecules. Finally, we identify key knowledge gaps and propose future research directions to facilitate clinical translation. A deeper understanding of PANoptosis in diabetic complications may pave the way for novel therapeutic approaches that simultaneously block multiple cell death pathways to ameliorate disease progression.

## 1 Introduction

The escalating global diabetes epidemic underscores the urgent need to investigate the pathogenesis of its complications. According to the 11th edition of the Diabetes Atlas (2025), recently published by the International Diabetes Federation, diabetes has become one of the most burdensome non-communicable diseases in human history. As of 2024, approximately 589 million adults aged 20–79 worldwide were affected by diabetes, corresponding to a prevalence of 11.1% (1). More concerning is that, with the accelerating global population ageing, this figure is projected to reach 853 million by 2050, accounting for 12.96% of the adult population (1).

Chronic low-grade inflammation driven by the innate immune system is a core mechanism underlying the onset and progression of diabetes (2). In the diabetic milieu, immune cells and metabolic tissues not only sense endogenous damage-associated molecular patterns (DAMPs), such as advanced glycation end products, free fatty acids, and high concentrations of glucose (3, 4); they also recognise pathogen-associated molecular patterns (PAMPs), such as bacterial lipopolysaccharide (LPS) and viral nucleic acids (5, 6). These DAMPs and PAMPs can be recognised by various pattern recognition receptors, such as Toll-like receptors (TLRs), NOD-like receptors (NLRs), RIG-I-like receptors, and absent in melanoma 2 (AIM2) (5, 6). Upon binding to their respective ligands, pattern recognition receptors activate downstream signalling cascades, promoting the release of inflammatory cytokines and triggering various forms of programmed cell death (PCD), including pyroptosis, apoptosis, and necroptosis (5, 6).

Historically, pyroptosis, apoptosis, and necroptosis were generally regarded as independent pathological events (3, 7). However, growing evidence suggests that under metabolic stress conditions, such as hyperglycaemia, lipotoxicity, and advanced glycation end products, there is extensive crosstalk among these three PCD pathways (8, 9). In recent years, this crosstalk has been systematically elucidated and integrated into a new concept—PANoptosis (10). It is an inflammatory lytic cell death pathway initiated by specific innate immune sensors and precisely regulated by the PANoptosome, a megacomplex (11, 12). Currently, research on PANoptosis is primarily focused on host defence, infectious diseases, and cancer (12, 13), consistent with its role as a core component of the innate immune system. However, given that diabetes and its complications are essentially chronic inflammatory diseases driven by metabolic dysregulation, in which persistent endoplasmic reticulum stress (ERS), mitochondrial dysfunction, and the activation of inflammatory signals overlap significantly with the triggers of PANoptosis (14, 15), an in-depth exploration of the role of PANoptosis in the field of diabetes undoubtedly holds significant scientific value and clinical translational potential.

The common pathological basis of diabetic complications is chronic inflammation, oxidative stress (OS), and various forms of cell death triggered by persistent hyperglycaemia and metabolic disturbances (14, 15). As recently comprehensively reviewed, a multitude of inflammatory mediators directly drive the progression of these systemic complications, positioning anti-inflammatory strategies as a critical gateway for developing novel effective treatments (2). PCD is a key mechanism underlying diabetes-related tissue damage, and modulating specific PCD pathways can partially attenuate disease progression (16–18). However, clinical and preclinical studies indicate that targeting a single PCD pathway (e.g., pyroptosis or apoptosis alone) has yielded limited therapeutic benefits in certain models of diabetic complications, which may be attributed to extensive crosstalk among PCD pathways (7, 19). Therefore, although research on PANoptosis in metabolic diseases is still in its infancy, in-depth exploration of its mechanisms in diabetes and its complications holds promise for identifying novel therapeutic strategies and drug targets.

This review summarises the core molecular mechanisms of PANoptosis and highlights recent advances in the field. It focuses on the mechanisms underlying pyroptosis, apoptosis, and necroptosis under diabetic metabolic stress, the complex crosstalk among these three processes, and the potential of targeting PANoptosis as a novel therapeutic strategy. Furthermore, we delve into the potential role of PANoptosis in various diabetic complications, including diabetic kidney disease (DKD), diabetic retinopathy (DR), diabetic neuropathy (DN), diabetic cardiomyopathy (DCM), and diabetic foot ulcer (DFU). More importantly, based on our analysis of the assembly and regulatory mechanisms of the PANoptosome complex, we identify potential therapeutic targets for intervening in diabetes and its complications, aiming to provide a theoretical basis and direction for the development of novel therapies.

## 2 Programmed cell death

As a genetically regulated form of PCD, PCD not only plays a role in the development and maintenance of homeostasis but also exerts a central role in various metabolic diseases (4, 20). Depending on whether cell membrane integrity is disrupted and whether an inflammatory response is triggered, PCD can be classified into two major categories: lytic cell death and non-lytic cell death (4, 7). Lytic cell death (such as pyroptosis, necroptosis and PANoptosis) is accompanied by cell membrane rupture and the release of large quantities of DAMPs and pro-inflammatory cytokines such as interleukin-1β (IL-1β) and interleukin-18 (IL-18), driving a vigorous inflammatory response; in contrast, non-lytic cell death (such as apoptosis) silently eliminates cells through the formation of apoptotic bodies, thereby avoiding the activation of inflammation (4, 7). However, this traditional paradigm has been expanded; apoptosis can transition into a lytic and inflammatory form (secondary pyroptosis) via the cleavage of gasdermins, such as gasdermin E (GSDME), orchestrated by classical apoptotic caspases.

### 2.1 Apoptosis

Apoptosis is characterised by cell shrinkage, chromatin condensation, DNA fragmentation, and the formation of apoptotic bodies; this process typically does not trigger a significant inflammatory response. The caspase family primarily executes apoptosis and can be activated via multiple pathways, including the intrinsic mitochondrial pathway and the extrinsic death receptor pathway (21, 22).

**Figure 1:**
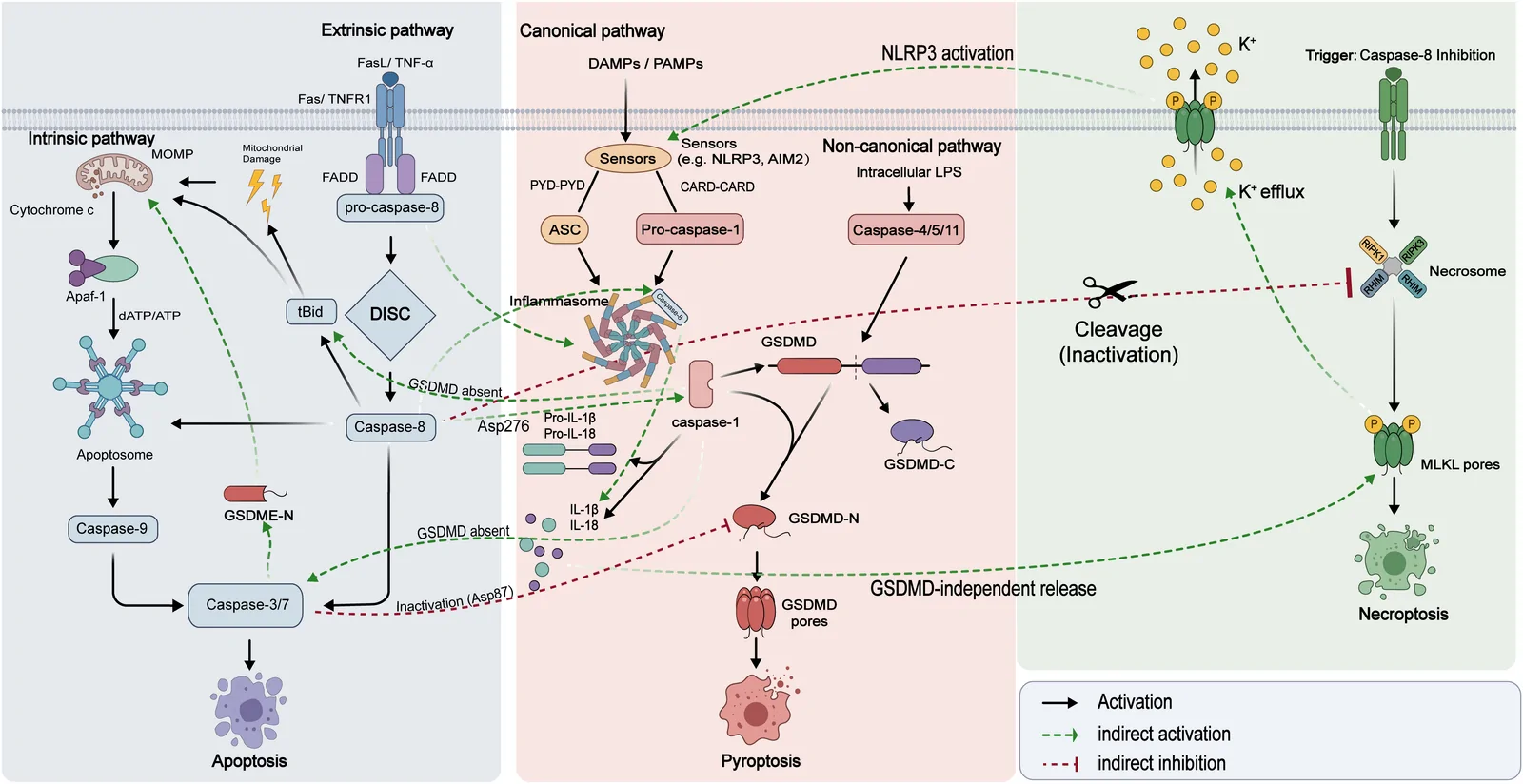
Crosstalk among apoptosis, pyroptosis, and necroptosis. Apaf-1, apoptotic protease-activating factor 1; ASC, apoptosis-associated speck-like protein containing a CARD; DAMP, damage-associated molecular pattern; DISC, death-inducing signalling complex; FADD, Fas-associated death domain protein; Fas, Fas receptor; FasL, Fas ligand; GSDMD, gasdermin D; GSDME, gasdermin E; IL-1β, interleukin-1β; IL-18, interleukin-18; LPS, lipopolysaccharide; MLKL, mixed-lineage kinase-like protein; MOMP, mitochondrial outer membrane permeabilization; NLRP3, NOD-like receptor family pyrin domain-containing protein 3; PAMP, pathogen-associated molecular pattern; RHIM, RIP homotypic interaction motif; RIPK1, receptor-interacting protein kinase 1; RIPK3, receptor-interacting protein kinase 3; tBid, truncated Bid; TNF-α, tumour necrosis factor-alpha; TNFR1, tumour necrosis factor receptor 1.

The intrinsic pathway is triggered by intracellular stressors such as DNA damage and growth factor deprivation. These signals upregulate or activate BH3-only proteins, thereby disrupting the homeostatic balance between anti-apoptotic and pro-apoptotic B-cell lymphoma 2 family proteins (23). This disruption of homeostasis ultimately leads to mitochondrial outer membrane permeabilisation, prompting the release of pro-apoptotic factors, such as cytochrome c, from the mitochondrial intermembrane space into the cytoplasm (24, 25).In the cytoplasm, cytochrome c binds to apoptotic protease-activating factor 1 and, in the presence of dATP/ATP, assembles into an apoptosome, which subsequently activates caspase-9 and initiates the caspase cascade (26). The extrinsic pathway, in contrast, is triggered by the binding of extracellular death ligands, such as Fas ligand (FasL) and tumour necrosis factor-alpha (TNF-α), to their respective receptors. This binding leads to the recruitment of the adaptor protein Fas-associated death domain protein (FADD) and the caspase-8 precursor to the death domain of the receptor’s intracellular domain, forming a death-inducing signalling complex, which subsequently initiates the self-activation of caspase-8 (27, 28). Activated caspase-8 can directly cleave and activate downstream effector caspases, caspase-3 and caspase-7, thereby executing the apoptotic programme. Furthermore, caspase-8 can cleave BH3-interacting domain death agonist (Bid), a BH3-only protein, generating its active form, truncated Bid. Truncated Bid subsequently translocates to the mitochondria, where it amplifies endogenous apoptotic signals by promoting mitochondrial outer membrane permeabilisation, thereby linking the two major apoptotic pathways (29, 30) (**Figure 1**).

### 2.2 Pyroptosis

Pyroptosis is a form of programmed necrotic cell death mediated by proteins of the Gasdermin family and accompanied by a strong inflammatory response; its classical pathway relies on the activation of inflammatory caspases (primarily caspase-1) (31).In this pathway, inflammasomes, represented by NLRP3, act as intracellular multiprotein complexes. Upon recognition of DAMPs or PAMPs by sensor proteins (such as NLRP3), these signals recruit the adaptor protein apoptosis-associated speck-like protein containing a caspase recruitment domain (CARD) (ASC) and trigger assembly, thereby activating caspase-1 (32, 33). Activated caspase-1 cleaves and activates the pro-inflammatory cytokine precursors pro-IL-1β and pro-IL-18, driving a robust inflammatory response; simultaneously, it specifically cleaves gasdermin D (GSDMD), releasing the N-terminal domain of GSDMD (31). The N-terminal domain of GSDMD subsequently oligomerises and forms pores in the cell membrane, directly leading to osmotic swelling, rupture, and the release of cellular contents—the execution phase of pyroptosis (34, 35). As research has progressed, our understanding of pyroptosis has moved beyond the classical caspase-1-dependent paradigm. Further studies have shown that caspases-4, 5, and 11 can be directly activated by intracellular LPS, which subsequently cleave GSDMD; this non-classical inflammasome pathway can also effectively induce pyroptosis (36–38) (**Figure 1**).

### 2.3 Necroptosis

Necroptosis is a form of caspase-independent PCD. Typical morphological features of this process include marked cell swelling and plasma membrane rupture (39). The classical pathway of necroptosis is triggered by death receptors (such as tumour necrosis factor receptor 1 and Fas) upon ligand stimulation. Its core molecular events depend on the interaction between receptor-interacting protein kinases 1 and 3 (RIPK1 and RIPK3) via their RIP homotypic interaction motif (RHIM) domains, forming an amyloid-like signalling complex known as the ‘necrosome’. The necrosome subsequently recruits and phosphorylates its key substrate, mixed-lineage kinase-like protein (MLKL) (40, 41). Activated MLKL oligomerises and translocates to the plasma membrane, directly disrupting membrane integrity and ultimately leading to cell death (39) (**Figure 1**).

## 3 Crosstalk between pyroptosis, apoptosis, and necroptosis

### 3.1 Apoptosis and pyroptosis

There is a complex interplay between pyroptosis and apoptosis. Taabazuing et al., through studies using dipeptidyl peptidase 8/9 inhibitors to activate caspase-1, found that in the absence of GSDMD, activated caspase-1 alternatively activates caspase-3 and -7, thereby inducing cells to undergo apoptosis. This finding clearly demonstrated, for the first time, that GSDMD is a key molecular switch that diverts cell fate towards pyroptosis (42). Subsequently, Tsuchiya et al. further validated this phenomenon in various models, including GSDMD-deficient bone marrow-derived macrophages and L929 cells. Their mechanistic studies revealed that following GSDMD knockout, caspase-1 cleaves Bid to generate truncated Bid, which in turn triggers the release of mitochondrial cytochrome c and the activation of caspase-9, ultimately initiating the intrinsic mitochondrial apoptosis pathway (43). Collectively, these studies reveal that when the pyroptosis pathway is blocked, cells switch to the apoptosis pathway as a backup mechanism. In addition to the shift from pyroptosis to apoptosis following pyroptosis inhibition, recent research has also revealed that the apoptosis pathway actively regulates pyroptosis. Demarco et al. demonstrated that the apoptosis-initiating caspase-8 can directly cleave GSDMD (at the human D276 site), thereby triggering pyroptosis, suggesting that apoptosis can also initiate pro-inflammatory cell death (44). However, the interaction between these two pathways is not a simple unidirectional activation, but rather exhibits complex synergistic and inhibitory effects. For example, in an osteomyelitis model, the NLRP3 inflammasome and caspase-8 exhibit functional redundancy, with both promoting the maturation and release of IL-1β, illustrating the synergistic interaction between the apoptotic and pyroptotic pathways under specific pathological conditions (45). Conversely, in cell models such as HEK 293T, THP-1, and RAW 264.7, it has been found that activated executor caspases (caspase-3/7) can specifically cleave the Asp87 site of human GSDMD, producing an inactive p43 fragment, thereby blocking the occurrence of pyroptosis (42). This negative regulation reveals that, whilst executing its classical death programme, apoptosis simultaneously possesses a ‘braking’ mechanism that inhibits inflammatory cell lysis.

In addition to interactions at the cell membrane, crosstalk between pyroptosis and apoptosis is also precisely regulated at the mitochondrial level. Rogers et al. found that the N-terminal domain of GSDME fragment, generated by caspase-3 cleavage, can directly target the mitochondrial membrane, releasing cytochrome c through pore formation, thereby amplifying caspase-3 activation and forming a positive feedback loop (46). This study also suggests that the N-terminal domain of GSDMD possesses the potential to target mitochondria, implying that this mechanism may have broader biological significance (47). Furthermore, the cascade of reactions involving inflammasomes and apoptosis-related molecules constitutes another key link between pyroptosis and apoptosis. For example, Lamkanfi’s team used omics technologies to identify caspase-7 as a direct substrate of caspase-1, thereby revealing that the NLRP3/caspase-1/caspase-7 signalling axis mediates inflammasome-driven apoptosis during Salmonella infection (48). In parallel, research by Gurung et al. established a direct link between the death receptor signalling pathway and the inflammatory response; they found that FADD and caspase-8 are not only key molecules in the apoptotic pathway but also essential nodes for the activation of the NLRP3 inflammasome (49). This discovery provides direct molecular evidence of how apoptosis participates in and regulates the inflammatory response (**Figure 1**).

### 3.2 Apoptosis and necroptosis

Caspase-8 is not only the initiator of extrinsic apoptosis, but also the absolute ‘gatekeeper’ of immune homeostasis and suppressor of necroptosis (50). Under physiological conditions or following simple apoptosis induction, activated caspase-8, through heterodimerisation, specifically cleaves and inactivates RIPK1 and RIPK3, thereby potently inhibiting the formation of the necrosome and the oligomerisation of MLKL. However, under conditions of diabetes-associated viral infection or extreme metabolic stress (such as severe ERS leading to impaired caspase-8 activity), this inhibitory ‘brake’ is released. Subsequently, RIPK1 and RIPK3 bind to each other via their characteristic RHIM, undergo liquid-liquid phase separation-mediated amyloid fibrillation and aggregation, phosphorylate the downstream effector MLKL, thereby committing the cell irreversibly to pro-inflammatory necroptosis. This explains why, in models such as DCM, the simple inhibition of caspase-mediated apoptosis may actually trigger a more intense inflammatory storm (51) (**Figure 1**).

### 3.3 Pyroptosis and necroptosis

Pyroptosis and necroptosis not only share downstream inflammatory effectors but also exhibit an independent cross-activation mechanism. Recent studies have shown that MLKL, the executioner protein of necroptosis, can directly trigger the release of IL-1β within the pyroptosis pathway, and this process is independent of GSDMD, a key effector protein in pyroptosis (52). Specifically, researchers used small-molecule ligands to activate MLKL and found that MLKL activation is sufficient to disrupt cell membrane integrity directly, leading to potassium ion efflux from the cell. This ionic disturbance, in turn, independently activates the NLRP3 inflammasome, promoting caspase-1-mediated IL-1β maturation and ultimately facilitating IL-1β release via membrane rupture induced by MLKL. Notably, this process proceeds normally in GSDMD-deficient cells, indicating that necroptosis can drive inflammatory responses via a mechanism entirely independent of the classical pyroptosis pathway (53) (**Figure 1**).

## 4 PANoptosis

PANoptosis is a novel concept systematically proposed by the Kanneganti laboratory in 2019, emerging from the understanding that these complex networks of positive synergies and negative constraints indicate significant compensatory capacity and functional redundancy among PCD pathways in metabolic diseases (54–56). The name ‘PAN’ derives from the three modes of cell death it integrates: Pyroptosis, Apoptosis, and Necroptosis (54).

PANoptosis is a unique innate immune inflammatory lytic cell death pathway, initiated by innate immune sensors and driven by caspases and RIPKs (54). The core mechanism underlying PANoptosis lies in the assembly of the PANoptosome complex, which provides a synergistic molecular scaffold for key molecules involved in pyroptosis, apoptosis, and necroptosis, thereby activating downstream effector molecules to induce lytic cell death (54). Currently, six main types of PANoptosomes have been identified: the Z-DNA-binding protein 1 (ZBP1)-PANoptosome, the AIM2-PANoptosome, the RIPK1-PANoptosome, the NLRP12-PANoptosome, as well as the more recently defined NLRP3-PANoptosome and NLRC5-PANoptosome.

### 4.1 ZBP1-PANoptosome

ZBP1 is a cytoplasmic nucleic acid sensor that specifically recognises Z-nucleic acids (57). ZBP1 contains two N-terminal Zα domains (Zα and Zβ) for recognising Z-nucleic acids, as well as two RHIMs that mediate interactions with downstream molecules (58, 59).

ZBP1-PANoptosome was first elucidated in detail in a model of influenza A virus infection (53). Influenza A virus infection activates the type I interferon signalling pathway, which upregulates ZBP1 expression via the RIG-I-MAVS axis; subsequently, ZBP1 detects virus-generated Z-RNA via its Zα domains, triggering conformational activation (59, 60). Activated ZBP1 interacts with RIPK3 via its RHIM domain, thereby recruiting RIPK1; the latter, through its death domain, recruits FADD, which in turn further recruits molecules such as caspase-8, caspase-6, ASC, NLRP3, and caspase-1, assembling into the ZBP1-PANoptosome (59). Studies have shown that caspase-6 plays a facilitating role in the assembly of this complex: caspase-6 binds to RIPK3 via its intrinsic disordered region, enhancing the RHIM-dependent interaction between ZBP1 and RIPK3, thereby promoting the assembly and activation of the PANoptosome; this function is independent of its proteolytic activity.

The ZBP1-PANoptosome is subject to multilayered regulation. RIPK1 competes with ZBP1 for binding to RIPK3 via its RHIM domain, thereby inhibiting the initiation of PANoptosis—an important negative feedback mechanism that maintains cellular homeostasis (59). ADAR1 interacts directly with ZBP1 via its Zα domain, thereby interfering with the formation of the ZBP1-RIPK3 complex and suppressing PANoptosis. During influenza A virus infection, ZBP1 undergoes critical ubiquitination, which further promotes its recognition of viral ribonucleoprotein complexes and signal transduction (59). Recent studies have revealed that the Zαβ domain of ZBP1 forms aggregates with liquid-liquid phase separation properties upon binding to Z-nucleic acids, promoting ZBP1 oligomerisation and downstream signal transduction; whereas full-length ZBP1 forms amyloid aggregates, which polymerise with RIPK3 via RHIM-RHIM interactions, driving necroptosis (58).

**Figure 2:**
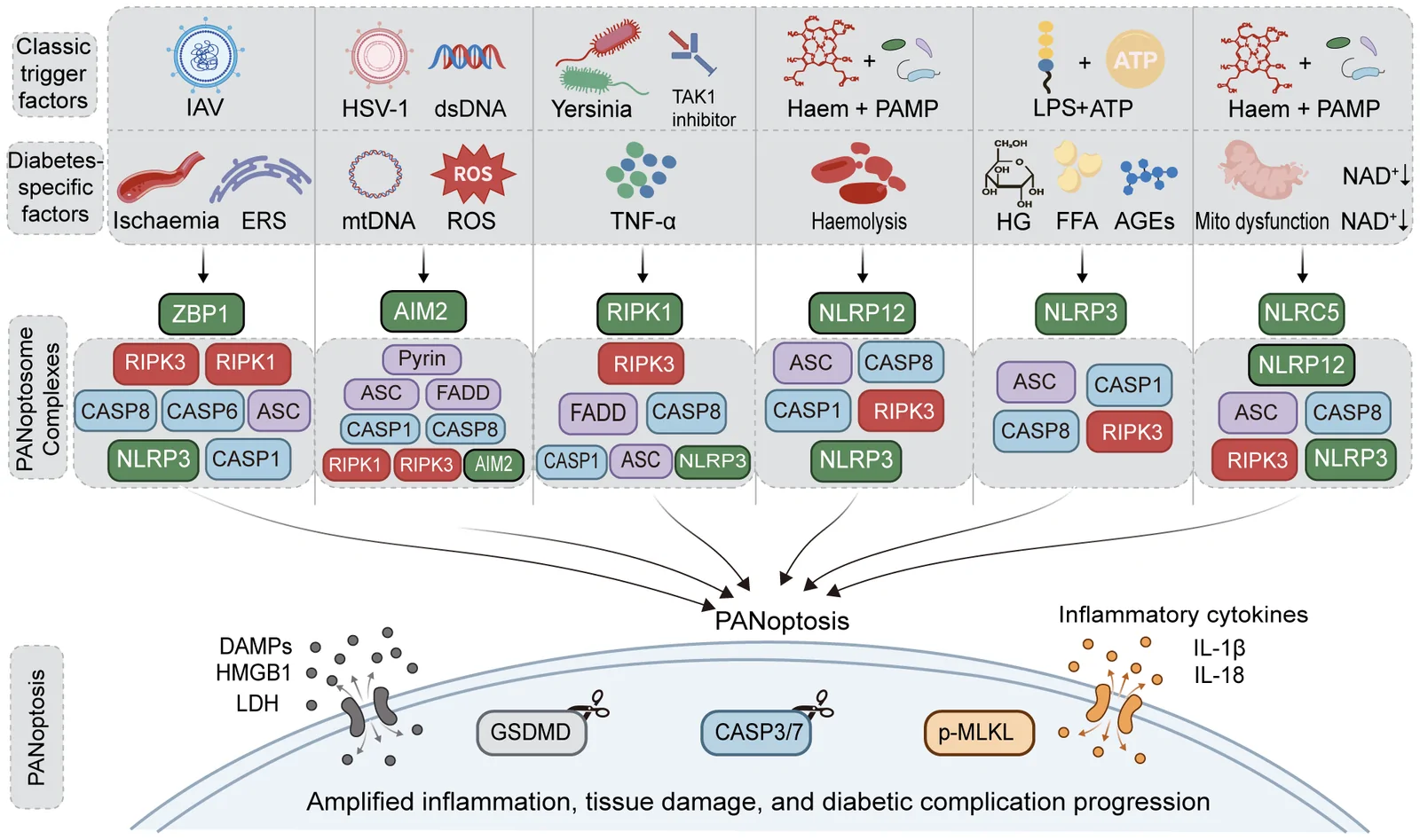
Schematic of PANoptosome assembly and its contribution to diabetic complications. Above the dashed line are canonical triggers of PANoptosome formation, while below the dashed line are diabetes-specific pathological triggers. These stimuli drive the assembly of diverse PANoptosome complexes, which execute PANoptosis to promote inflammatory amplification, tissue damage, and diabetic complication progression. AIM2, absent in melanoma 2; AGEs, advanced glycation end products; dsDNA, double-stranded DNA; ERS, endoplasmic reticulum stress; FFA, free fatty acids; HG, high glucose; HMGB1, high mobility group box 1; HSV-1, herpes simplex virus type 1; IAV, influenza A virus; LDH, lactate dehydrogenase; Mito, mitochondrial; mtDNA, mitochondrial DNA; NAD^+^, nicotinamide adenine dinucleotide; ROS, reactive oxygen species; TAK1, transforming growth factor-beta-activated kinase 1; ZBP1, Z-DNA-binding protein 1. Parts of this figure were created using assets from BioGDP (https://BioGDP.com) (78).

In DFU and DCM, ischaemia-reperfusion injury and ERS can lead to the release of endogenous nucleic acids. This may link cellular stress to inflammatory cell death by activating the ZBP1-PANoptosome, providing a new perspective on understanding the initiation mechanisms of cell death in these complications (**Figure 2**).

### 4.2 AIM2-PANoptosome

AIM2 is a member of the PYHIN family, comprising an N-terminal pyrin domain and a C-terminal HIN domain. As a classic cytoplasmic DNA sensor, AIM2 can directly recognise double-stranded DNA (dsDNA); this recognition is not sequence-dependent but occurs via the HIN domain binding to the DNA sugar-phosphate backbone.

The AIM2-PANoptosome was first identified in models of herpes simplex virus type 1 and Francisella infection. Studies have shown that, following the detection of cytoplasmic dsDNA, AIM2 undergoes homotypic interaction with the pyrin domain of ASC via its own pyrin domain, thereby recruiting ASC to form an oligomerisation platform. Unlike the classical AIM2 inflammasome, the AIM2-PANoptosome further integrates additional death-related molecules: whilst ASC recruits caspase-1 via its CARD domain, it also recruits caspase-8 via FADD; furthermore, RIPK1, RIPK3, ZBP1, and Pyrin have been confirmed as key components of this complex (12, 61). Consequently, the typical composition of the AIM2-PANoptosome includes AIM2, ASC, caspase-1, caspase-8, FADD, RIPK1, RIPK3, ZBP1, and Pyrin (12). The assembly of this multi-protein complex provides the structural basis for the simultaneous activation of three cell death pathways: caspase-1 cleaves GSDMD to drive pyroptosis, caspase-8 activates downstream caspase-3 to mediate apoptosis, and RIPK3 phosphorylates MLKL to trigger necroptosis (10, 56).

The AIM2-PANoptosome is subject to multilayered regulation. Interferon regulatory factor 1 (IRF1) has been identified as a key upstream transcriptional regulator of this complex. Studies indicate that, upon pathogen stimulation, IRF1 promotes the efficient assembly of the PANoptosome by inducing the expression of AIM2 and related components; IRF1 deficiency significantly impairs AIM2-PANoptosome activation and subsequent PANoptosis (62). Furthermore, AIM2 synergistically interacts with other sensors, including ZBP1 and Pyrin, to jointly drive inflammatory cell death signalling (61).

As a DNA sensor, AIM2 is activated by mitochondrial or nuclear DNA damage in DKD and DCM, strongly suggesting that the AIM2-PANoptosome may play a key role in linking metabolic stress (such as mitochondrial dysfunction) to inflammatory cell death in these complications (**Figure 2**).

### 4.3 RIPK1-PANoptosome

RIPK1 is a multi-domain protein of approximately 76 kDa that occupies a pivotal position in the cellular death and inflammatory signalling networks. RIPK1 regulates catalytic activity via its N-terminal kinase domain; the intermediate RHIM domain mediates interactions with proteins such as RIPK3, whilst the C-terminal death domain is responsible for recruiting death domain-containing adaptor proteins such as FADD (63). This multi-domain architecture confers a dual function on RIPK1 in the regulation of cell death: acting as a kinase to initiate downstream signalling, whilst simultaneously functioning as a scaffold protein involved in the assembly of multi-protein complexes.

The RIPK1-PANoptosome was first identified in a Yersinia infection model. Studies have shown that Yersinia infection induces the formation of a RIPK1-containing multimolecular complex in macrophages, comprising key molecules such as RIPK1, FADD, caspase-8, ASC, NLRP3, RIPK3, and caspase-1, which can simultaneously regulate pyroptosis, apoptosis, and necroptosis (63, 64). Further studies have revealed that, under conditions of transforming growth factor-beta-activated kinase 1 (TAK1) deficiency or inhibition, combined TLR stimulation can similarly induce the formation of the RIPK1-PANoptosome (13, 64).

The RIPK1-PANoptosome is subject to precise regulation. cIAP1/2 are key E3 ubiquitin ligases for RIPK1 that maintain RIPK1 stability by mediating its ubiquitination. Inhibition of cIAP1/2 reduces RIPK1 phosphorylation and ubiquitination, thereby exacerbating inflammatory responses and PANoptosis. Furthermore, a genome-wide CRISPR screen revealed that RNA-binding proteins such as RAVER1 and PTBP1 can precisely control TAK1 inhibitor-induced PANoptosis by regulating the alternative splicing of RIPK1 (61, 64). IRF1 has been identified as an upstream transcriptional regulator of the RIPK1-PANoptosome, promoting RIPK1-mediated PANoptosis in response to TAK1 inhibition and LPS stimulation (62).

As the core scaffolding molecule of the PANoptosome, RIPK1 has been identified as a key therapeutic target in various complications, including DKD and DCM. Targeting RIPK1 kinase activity or scaffolding function may simultaneously block multiple cell death pathways by disrupting PANoptosome assembly, providing a theoretical basis for the development of broad-spectrum cell death inhibitors (**Figure 2**).

### 4.4 NLRP12-PANoptosome

NLRP12 is a unique cytoplasmic pattern recognition receptor within the NLR family. The function of NLRP12 has long been a subject of debate; previous studies have largely focused on its role in negatively regulating inflammatory signalling, such as by attenuating inflammatory responses through the inhibition of the NF-κB and MAPK pathways (11, 65). However, recent studies have revealed that, under specific stimuli, NLRP12 acts as a core sensor to assemble into a PANoptosome, thereby driving PANoptosis. This functional shift from a negative regulator of inflammation to a pro-inflammatory death sensor highlights the complexity and context-dependence of NLRP12 as a ‘dual-function’ molecule (11).

The NLRP12-PANoptosome was first systematically elucidated in models involving haem combined with PAMP or TNF stimulation (11, 66). Studies have shown that synergistic stimulation by haem and PAMPs (such as LPS and Pam3CSK4) or TNF-α induces the formation of an NLRP12-containing multimolecular complex in macrophages. The typical components of this complex include NLRP12 (sensor), NLRP3, ASC (adaptor protein), caspase-1, caspase-8, and RIPK3 (11, 66).

The NLRP12-PANoptosome is subject to precise regulation at multiple levels. TLR2/4 signalling induces upregulation of NLRP12 expression via IRF1, providing the components necessary for PANoptosome assembly (11, 66). Recent studies have revealed that haematopoietic cell kinase (HCK) is a key regulatory molecule of the NLRP12-PANoptosome (67). HCK is significantly upregulated in haemolytic disease samples and in macrophages co-stimulated with haem and TNF; knocking down HCK inhibits NLRP12-mediated PANoptosis. In macrophages stimulated with haem and PAMPs, NLRP12 expression is significantly upregulated 36 hours post-stimulation, which closely coincides with the onset of cell death (30–32 hours), suggesting that NLRP12 induction is a key upstream event in the initiation of PANoptosis (11).

Haem is released in large quantities in complications such as DFU due to microvascular damage and haemolysis; when combined with PAMPs released from infection sources, this can synergistically activate the NLRP12-PANoptosome. This may explain the mechanism underlying the formation of the inflammatory microenvironment that hinders wound healing in DFU and provides new insights for targeting this pathway to improve wound healing (**Figure 2**).

### 4.5 NLRP3-PANoptosome

NLRP3 is the most extensively studied cytoplasmic pattern recognition receptor within the NLR family and has long been regarded as a core component of the classical inflammasome, mediating pyroptosis via caspase-1 activation (32, 33). However, a recent study by Sharma et al. revealed that under classical inflammasome activation conditions—such as co-stimulation with LPS and ATP or LPS and nigericin—NLRP3 functions as a core sensor by assembling into a PANoptosome, driving integrated PANoptosis. This study demonstrates that even in caspase1⁻/⁻ or GSDMD⁻/⁻ cells, NLRP3 can still form a multi-protein complex by recruiting caspase8 and RIPK3, inducing delayed cell death characterised by pyroptotic, apoptotic, and necroptotic features. Immunofluorescence colocalisation confirmed that formation of the PANoptosome complex, comprising NLRP3, ASC, CASP8, and RIPK3, was observed within 30 minutes of stimulation. Genetic evidence demonstrated that Casp1⁻/⁻Casp8⁻/⁻Ripk3⁻/⁻ cells were completely resistant to LPS and ATP-induced cell death, confirming that this cell death is indeed PANoptosis. The typical composition of the NLRP3-PANoptosome includes NLRP3, ASC, caspase1, caspase8, and RIPK3. This discovery extends the function of NLRP3 beyond the regulation of pyroptosis to a broader integrated network of inflammatory cell death, offering novel insights into the pathological mechanisms underlying NLRP3-associated inflammatory diseases (68).

As a central sensor of metabolic stress, NLRP3 is robustly activated by diabetes-related stimuli, such as hyperglycaemia, free fatty acids, and uric acid crystals. Its involvement in the pathogenesis of DKD, DR, and DCM is well established. The recently identified NLRP3-PANoptosome mechanism suggests that NLRP3 may directly link metabolic dysregulation to integrated cell death via PANoptosome assembly in these complications, thereby providing a more robust theoretical foundation for targeting NLRP3 in the intervention of multiple complications (**Figure 2**).

### 4.6 NLRC5-PANoptosome

NLRC5 is a relatively enigmatic member of the NLR family, traditionally regarded as functioning primarily as a transcriptional activator for MHC class I molecules, shuttling between the nucleus and the cytoplasm (69). However, a recent study by Sundaram et al. has revealed that, under specific stimulatory conditions, NLRC5 can act as an innate immune sensor, assembling into an NLRC5-PANoptosome to drive PANoptosis. Via systematic screening, the study found that NLRC5 responds specifically to combined stimulation with haem and PAMPs (such as LPS, Pam3, R848) or haem and TNF, whilst showing no response to classical inflammasome agonists. Under haem and PAMP stimulation, intracellular nicotinamide adenine dinucleotide (NAD^+^) levels are depleted, leading to upregulation of NLRC5 expression via the TLR2/4 signalling pathway and promotion of reactive oxygen species (ROS) production; together, these mechanisms drive cell death. Immunofluorescence and co-immunoprecipitation confirmed that NLRC5 forms a multi-protein complex with NLRP12, ASC, CASPASE8, RIPK3, and NLRP3, with NLRC5 and NLRP12 serving as core components, whilst NLRP3 is primarily responsible for caspase1 activation but dispensable for cell death. Functional studies indicate that this complex can simultaneously activate pyroptosis (GSDMD/E cleavage), apoptosis (caspase3/caspase7 cleavage), and necroptosis (MLKL phosphorylation). In *in vivo* models, NLRC5⁻/⁻ mice exhibited significant protective effects against haemolytic disease, colitis, and haemophagocytic lymphohistiocytosis, with markedly reduced inflammatory responses, tissue damage, and mortality (70). The identification of the NLRC5-PANoptosome uncovers a novel role for NLRC5 as a ‘dual-function’ molecule—acting both as an MHC class I transcriptional regulator and as a cytoplasmic sensor of NAD^+^ depletion—linking metabolic stress to inflammatory cell death and providing a new theoretical basis for understanding the mechanisms of cell death in haemolytic-related diseases and states of metabolic dysregulation (70).

As a sensor of NAD^+^ depletion, NLRC5 activation is closely associated with energy metabolic dysregulation in diabetic complications. In diabetic complications, such as cardiomyopathy and nephropathy, mitochondrial dysfunction and NAD^+^ depletion are central pathological events (14, 71, 72). This strongly implicates the NLRC5-PANoptosome in directly linking metabolic stress to inflammatory cell death in these diseases, offering a novel molecular link that bridges the ‘metabolic-inflammatory-death’ axis in diabetic complications (**Figure 2**).

### 4.7 Immuno-regulatory dimensions of PANoptosis

Emerging evidence indicates that immune cells not only undergo PANoptosis but also actively shape pro-PANoptotic microenvironments through paracrine signals and effector molecules. In diabetic periodontitis, M1-polarized macrophages simultaneously exhibit cleaved caspase-1, caspase-3, and p-MLKL, and signal transducer and activator of transcription 1 (STAT1) serves as a critical node integrating inflammatory polarization signals with PANoptosis execution (73). Cytotoxic lymphocyte-derived granzymes provide a direct molecular link between adaptive immunity and PANoptosis: granzyme A induces both apoptosis (SET complex disruption) and pyroptosis (gasdermin B cleavage), while granzyme B engages caspase-3 and GSDME pathways, with both predominantly expressed in immune cells and upregulated in diabetic kidneys (74, 75). T cell subsets exert opposing effects—CD4^+^ T cells and γδ T cells promote inflammatory signalling, whereas regulatory T cells correlate negatively with pyroptosis, potentially serving as an immunological “brake” on PANoptosis. NETosis-derived extracellular DNA may provide danger signals for AIM2/ZBP1-associated inflammatory pathways, linking innate immunity to PANoptosome assembly (76). Systems-level analyses further demonstrate quantitative correlations between PANoptosis hub genes and immune infiltration (77). Notably, current evidence primarily supports “PANoptosis-related marker co-activation” rather than definitive PANoptosome assembly in diabetic tissues; future studies require co-immunoprecipitation and genetic approaches to confirm complex formation. Collectively, these findings establish immuno-regulatory networks as an integral dimension of PANoptosis in diabetic complications, nominating STAT1, granzymes, and T cell subsets as potential therapeutic nodes.

## 5 PANoptosis in diabetic complications

### 5.1 Diabetic kidney disease

DKD is one of the most common microvascular complications of diabetes, clinically characterised by persistent proteinuria and progressive decline in renal function. It severely impacts patients’ quality of life and significantly increases the risk of end-stage renal disease and cardiovascular mortality (71, 79). PCD in renal cells is a central pathological mechanism mediating the onset and progression of DKD (80). Under hyperglycaemic conditions, metabolic disturbances ultimately trigger renal cell death, leading to loss of cellular function and disruption of tissue structure, thereby driving the progression of DKD (80). There is substantial evidence indicating that pyroptosis, apoptosis, and necroptosis are all closely associated with DKD. For example, in renal cells stimulated by high glucose, upregulation of RIPK3 and activation of the necroptosis signalling pathway can be observed (81). Concurrently, neutrophil extracellular traps can induce pyroptosis in glomerular endothelial cells (82). Furthermore, apoptosis, as a classical form of cell death, also plays a significant role in renal cell damage associated with DKD.

**Figure 3:**
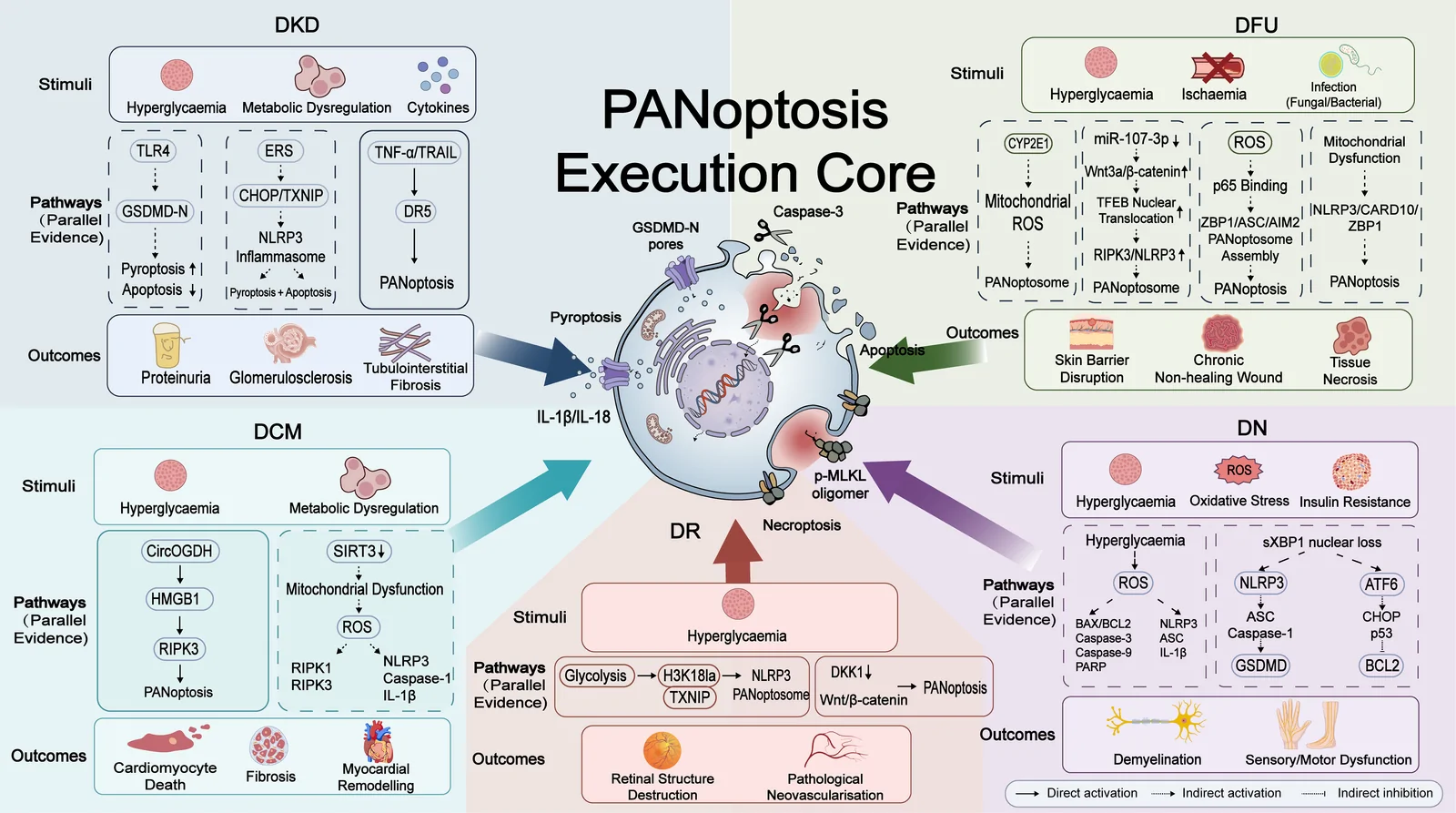
PANoptosis in diabetic complications: molecular mechanisms and pathological outcomes. This image systematically illustrates the cell death regulatory networks involved in common diabetic complications, including diabetic kidney disease (DKD), diabetic foot ulcer (DFU), diabetic cardiomyopathy (DCM), diabetic retinopathy (DR) and diabetic neuropathy (DN). Solid arrows represent direct stimulatory regulation, dashed arrows indicate indirect stimulatory crosstalk, and dashed T-shaped arrows denote indirect inhibitory regulation. Solid-line boxes mark well-established complete PANoptosis (co-activation of pyroptosis, apoptosis and necroptosis) directly verified in the corresponding diabetic complication. Dashed-line boxes in DKD, DCM, DR and DN refer to crosstalk between two of the three cell death pathways, lacking sufficient evidence for intact PANoptosis. For DFU, dashed-line boxes represent relevant cell death pathways confirmed in diabetes-related tissue injury models (e.g., limb ischaemia, diabetic skin flap, diabetic wound), which share highly overlapping pathological microenvironments (ischaemia, metabolic dysregulation, inflammation) with DFU and thus provide important mechanistic references for DFU research, as direct PANoptosis studies in DFU models are currently limited. BAX, Bcl-2-associated X protein; BCL2, B-cell lymphoma 2; CARD10, caspase recruitment domain family member 10; CHOP, C/EBP homologous protein; CYP2E1, cytochrome P450 2E1; DCM, diabetic cardiomyopathy; DFU, diabetic foot ulcer; DKD, diabetic kidney disease; DKK1, Dickkopf-1; DN, diabetic neuropathy; DR, diabetic retinopathy; DR5, death receptor 5; H3K18la, histone H3 lysine 18 lactylation; p53, tumour protein p53; p65, NF-κB subunit p65 (RELA); PARP, poly(ADP-ribose) polymerase; SIRT3, sirtuin 3; sXBP1, spliced X-box binding protein 1; TFEB, transcription factor EB; TLR4, Toll-like receptor 4; TXNIP, thioredoxin-interacting protein. Parts of this image were created using assets from BioGDP (https://BioGDP.com) (78).

Recent studies have provided compelling evidence for the concurrent activation of multiple PCD pathways in the development and progression of DKD, pointing toward the potential involvement of PANoptosis-like mechanisms. By integrating transcriptomic data from public databases and employing bioinformatic methods, Geng et al. identified three key genes (PDK4, YWHAH, and PRKX) and successfully established PANoptosis-associated genetic biomarkers for the diagnosis of DKD (83). In a streptozotocin-induced diabetic C57BL/6J mouse model, Lv et al. discovered through single-cell RNA sequencing that the TNF superfamily ligand TNF-related apoptosis-inducing ligand (TRAIL) and its receptor death receptor 5 (DR5) were significantly upregulated in podocytes. Further functional experiments demonstrated that TRAIL activates markers across all three PCD pathways in podocytes (such as cleaved caspase-1, cleaved caspase-3, and p-MLKL) via DR5. While this promotes podocyte damage and glomerulosclerosis and exhibits features consistent with PANoptosis, the physical assembly of a PANoptosome complex in this context remains to be directly visualised (84). Furthermore, although the studies by Cao et al. and Yuan et al. did not directly investigate PANoptosome assembly, they respectively revealed profound crosstalk between the ERS/NLRP3 inflammasome axis and the TLR4/GSDMD axis in regulating pyroptosis and apoptosis in DKD. Cao et al. found that the unfolded protein response simultaneously activates both NLRP3 inflammasome-mediated pyroptosis and apoptosis via the C/EBP homologous protein (CHOP)-thioredoxin-interacting protein (TXNIP) axis (85); Yuan et al., meanwhile, confirmed that GSDMD is a key molecule in the transition from apoptosis to pyroptosis, indicating that pyroptosis and apoptosis do not exist in isolation in DKD renal tubular epithelial cells, but rather coexist in a dynamic equilibrium at the molecular level (86). The coexistence and interaction of these multiple cell death pathways suggest the possible existence of a higher-order, comprehensive death programme mediated by the PANoptosome in DKD, providing important clues for further research in this field. In addition to Gasdermin proteins and caspases, the role of PANoptosis-associated receptors in DKD is also gradually attracting attention. Previous studies have confirmed that AIM2, acting as a DNA sensor, is up-regulated in DKD renal tubular epithelial cells and, by detecting ROS-induced DNA damage, recruits ASC and caspase-1 to drive pyroptosis and inflammatory responses (87).Although this study has not yet directly investigated whether AIM2 is involved in apoptosis or necroptosis, its structural and functional characteristics are highly similar to the AIM2 module in the PANoptosome, suggesting that it may possess broader potential for regulating cell death in DKD, warranting further investigation (**Figure 3**).

Targeting these integrated PCD networks has emerged as a highly promising new strategy for the treatment of DKD. Targeting upstream regulatory pathways to inhibit the activation of sensor molecules is an effective strategy. The use of soluble DR5-Fc to block the TRAIL/DR5 interaction significantly reduced the urine albumin-to-creatinine ratio in a BTBR ob/ob diabetic mouse model, improved glomerular hypertrophy and podocyte fusion, and suppressed the expression of multiple cell death markers in podocytes (84).RIPK1, as the core scaffold molecule of the PANoptosome, represents an important therapeutic target. Studies have shown that spermidine can inhibit RIPK1 kinase activity by mediating its acetylhypusination, thereby improving glucose metabolism disorders and vascular pathology in diabetic models (88). Furthermore, in the context of DKD, fatty acids can synergise with hyperglycaemia to induce necroptosis in renal tubular epithelial cells, whereas the application of the RIPK1 inhibitor RIPA-56 significantly alleviates renal injury and inhibits this pathway (89). As a key sensor, AIM2 drives pyroptosis and inflammation in a manner highly similar to that of the PANoptosome. Consequently, the search for targeted molecules or drugs that act on the AIM2 receptor represents another promising therapeutic strategy. Recently, Ullah et al. identified the lead compound NPA024823, which binds stably to AIM2, through computational virtual screening of natural product databases, providing a structural basis for the design of AIM2-targeting drugs (90). Further functional studies have shown that wogonin can inhibit the TLR4/Janus kinase/signal transducer and activator of transcription/signalling pathway by upregulating suppressor of cytokine signalling 3, thereby downregulating AIM2 inflammasome activation and alleviating high-glucose-induced damage to renal tubular epithelial cells and fibrotic responses (91). Collectively, these findings suggest that targeting different components of these interconnected cell death pathways represents a potential novel therapeutic approach for DKD (**Table 1**).

**Table 1:** Potential agents or strategies targeting PANoptosis in diabetic complications.

Targeting strategy	Agent/therapy	Molecular target(s)	Diabetic complication	Outcome	Model/species	Ref.
Upstream pathway inhibitors	2-deoxy-D-glucose	Hexokinase/Glycolysis	DR	Attenuated RPE cell PANoptosis; reduced OS; improved retinal function	*In vivo*: STZ-induced diabetic C57BL/6J mice *In vitro*: HG-treated ARPE-19 & murine RPE cells	(99)
Upstream pathway inhibitors	shTXNIP	TXNIP/NLRP3 axis	DR	Inhibited PANoptosome assembly; attenuated RPE cell PANoptosis; improved retinal function	*In vivo*: STZ-induced diabetic C57BL/6J mice *In vitro*: HG-treated ARPE-19 & murine RPE cells	(99)
Upstream pathway inhibitors	DKK1	Wnt/β-catenin pathway	DR	Reduced pathological neovascularization; inhibited PANoptosis	*In vivo*: STZ-induced diabetic SD rats *In vitro*: HG-treated HRMVECs & rat retinal Müller cells	(100)
Upstream pathway inhibitors	siRNA against circOGDH	circOGDH	DCM	Improved cardiac function; reduced myocardial fibrosis	*In vivo*: STZ/HFD-induced diabetic C57BL/6J mice & db/db mice *In vitro*: HG/PA-treated rat H9c2 cardiomyocytes	(115)
Upstream pathway inhibitors	CD38 deficiency	CD38	DCM	Inhibited cardiomyocyte pyroptosis and apoptosis; improved cardiac function	*In vivo*: HFD/STZ-induced diabetic C57BL/6J mice *In vitro*: HG/PA-treated rat H9c2 cardiomyocytes	(119)
Upstream pathway inhibitors	siEMP1	EMP1	DCM	Suppressed pyroptosis and apoptosis; alleviated OS and mitochondrial damage; enhanced cell viability	*In vitro*: HG-treated rat H9c2 cardiomyocytes	(120)
Upstream pathway inhibitors	Soluble DR5-Fc	TRAIL/DR5 pathway	DKD	Attenuated podocyte injury and glomerulosclerosis; reduced urinary albumin-to-creatinine ratio	*In vivo*: BTBR ob/ob diabetic mice *In vitro*: TNF-α-stimulated human podocytes	(84)
Upstream pathway inhibitors	3,4-dimethoxychalcone	miR-107-3p/Wnt3a/mTOR/TFEB	Diabetic flap ischaemia^a^	Restored autophagic flux; inhibited PANoptosis; promoted tissue survival	*In vivo*: Ischemic skin flap model in C57BL/6J, Caspase-1 KO, and diabetic db/db mice	(127)
Upstream pathway inhibitors	si-TAK1	TAK1-NF-κB (p65)	DFU	Inhibited PANoptosome assembly; reduced cell death; promoted angiogenesis	*In vivo*: Full-thickness skin wound model in diabetic db/db mice *In vitro*: HG-treated HaCaT, fibroblasts, and HUVECs	(129)
PANoptosis sensors	Wogonin	SOCS3/TLR4/JAK/STAT/AIM2 axis	DKD	Attenuated renal inflammation and fibrosis	*In vivo*: Diabetic db/db mice *In vitro*: HG-treated HK-2 cells	(91)
PANoptosis sensors	Lead compound NPA024823	AIM2 inflammasome	DKD	Potential inhibitory activity against AIM2 inflammasome	^b^In silico: Computational virtual screening and molecular dynamics simulation (Target: Human AIM2)	(90)
Scaffolding/regulatory molecules	Spermidine	RIPK1	DKD	Protected renal microvascular endothelial cells; inhibited vascular leakage, inflammation, and cell death	Clinical: Human vascular tissues and diabetic nephropathy renal biopsy tissues *In vivo*: Nat1 conditional KO mice (C57BL/6 background) *In vitro*: MEFs & HEK293T cells	(88)
Scaffolding/regulatory molecules	RIPA-56	RIPK1	DKD	Attenuated renal injury; inhibited necroptosis pathway	Clinical: Human DKD renal biopsy tissues *In vivo*: HFD-fed diabetic db/db mice *In vitro*: HG/PA-treated HK-2 cells	(89)
Scaffolding/regulatory molecules	RS67333	5-HT4R/RIPK3 (indirect)	DN	Reduced neuronal loss; improved motor function	*In vivo*: STZ-induced diabetic mice (WT and RIPK3⁻/⁻ C57BL/6)	(103)
Mitochondrial/ER homeostasis	Salvianolic acid B	ROS	DN	Inhibited high glucose-induced Schwann cell apoptosis and pyroptosis; protected peripheral nerves	*In vitro*: HG-treated RSC96 cells	(106)
Mitochondrial/ER homeostasis	*Pseudostellaria heterophylla* polysaccharides	CYP2E1	DFU/DLLI^a^	Promoted angiogenesis; inhibited endothelial cell PANoptosis	*In vivo*: STZ-induced diabetic C57BL/6 mice with lower limb ischemia (DLLI model) *In vitro*: HG-exposed HUVECs	(126)
Mitochondrial/ER homeostasis	Isorhynchophylline	sXBP1 nuclear translocation	DN	Simultaneously inhibited ERS, pyroptosis, and apoptosis; improved cognitive function	*In vivo*: STZ-induced diabetic C57BL/6 mice & diabetic db/db mice *In vitro*: HG-induced insulin-resistant rat PHAs and CTX TNA2 astrocytes	(109)
Metabolic reprogramming	PCL-EVs nanofibre scaffold	Mitochondrial metabolic reprogramming (NLRP3/CARD10/ZBP1)	Diabetic skin defect^a^	Downregulated PANoptosis-related gene expression; suppressed inflammation; accelerated wound healing	*In vivo*: Full-thickness excisional skin defect (anti-contracture) model in SD rats *In vitro*: Murine L929 cells & C57BL/6 BMSC-differentiated ECs	(128)

a Although this study was not directly conducted in DFU models, its elucidation of PANoptosis regulatory mechanisms is closely associated with the pathophysiological microenvironment of DFU (ischaemia, metabolic dysregulation, and inflammation), providing a theoretical basis and potential therapeutic targets for DFU treatment.

b “In silico” entries in the Model/Species column (e.g., the lead compound NPA024823) represent computational predictions only and require further biological validation.

Cell lines: HaCaT (human keratinocyte cell line); HEK293T (human embryonic kidney cell line); HK-2 (human kidney proximal tubular epithelial cell line); H9c2 (rat cardiomyocyte cell line); RSC96 (rat Schwann cell line). Animal strains: C57BL/6J, C57BL/6, db/db, BTBR ob/ob, SD (Sprague-Dawley), and knock-out (KO) strains are as indicated. CARD10, caspase recruitment domain family member 10; CD38, cluster of differentiation 38; circOGDH, circular RNA OGDH; CYP2E1, cytochrome P450 2E1; DCM, diabetic cardiomyopathy; DFU, diabetic foot ulcer; DKD, diabetic kidney disease; DLLI, diabetic lower limb ischemia; DN, diabetic neuropathy; DR, diabetic retinopathy; DR5, death receptor 5 (TNFRSF10B); EMP1, epithelial membrane protein 1; ERS, endoplasmic reticulum stress; HFD, high-fat diet; HG, high glucose; HRMVECs, human retinal microvascular endothelial cells; HUVECs, human umbilical vein endothelial cells; JAK, Janus kinase; KO, knockout; MEFs, mouse embryonic fibroblasts; mTOR, mammalian target of rapamycin; NF-κB, nuclear factor kappa-light-chain-enhancer of activated B cells; NLRP3, NOD-like receptor family pyrin domain-containing protein 3; OS, oxidative stress; PA, palmitic acid (or palmitate); PCL-EVs, polycaprolactone-extracellular vesicles; RPE, retinal pigment epithelial; ROS, reactive oxygen species; RIPK3, receptor-interacting protein kinase 3; SD, Sprague-Dawley; shTXNIP, short hairpin RNA targeting TXNIP; SOCS3, suppressor of cytokine signalling 3; STAT, signal transducer and activator of transcription; STZ, streptozotocin; TAK1, transforming growth factor-beta-activated kinase 1; TFEB, transcription factor EB; TLR4, Toll-like receptor 4; TNF-α, tumour necrosis factor-alpha; TRAIL, TNF-related apoptosis-inducing ligand; TXNIP, thioredoxin-interacting protein; WT, wild-type; 5-HT4R, 5-hydroxytryptamine receptor 4 (serotonin receptor 4).

In summary, metabolic stress signals in DKD synergistically drive parallel cell death programmes via the TRAIL/DR5 pathway, ERS, and mitochondrial dysfunction, making the targeted inhibition of these pathways, along with core sensors (e.g., NLRP3, AIM2) and scaffold molecules (e.g., RIPK1), a highly promising therapeutic strategy.

### 5.2 Diabetic retinopathy

DR is the most common microvascular complication of diabetes and is also a progressive neurovascular disease characterised by neuronal damage, vascular leakage, and glial cell activation (92). Pyroptosis, apoptosis, and necroptosis have all been shown to contribute to the pathogenesis of DR (93–95). These PCD pathways interact significantly within the pathological environment of DR. In a systematic review of retinal ganglion cell death mechanisms, Jiang et al. noted that PANoptosis plays a significant role in various retinal pathologies (such as glaucoma and ischaemia-reperfusion injury), with the NLRP3 inflammasome identified as a key PANoptosome sensor (96).This provides a theoretical basis for exploring the role of PANoptosome-mediated cell death in retinal neurodegenerative diseases such as DR.

PANoptosis and its associated PCD networks likely play a pivotal role in the pathogenesis of DR. Hyperglycaemia is the core inducer driving these cell death pathways in DR. At the human disease level, by integrating the Gene Expression Omnibus database with machine learning algorithms, studies have identified significant dysregulation of a group of PANoptosis-related hub genes, such as BEX2 and FASN, in diabetic retinal tissue; this provides robust omics evidence for the concurrent regulation of PCD-related networks in the progression of DR (97). Furthermore, NLRP3, a key sensor of PANoptosis, is significantly activated in DR by factors such as dysregulated glucose metabolism (98). Research by Xi et al. further elucidated the precise molecular pathway. Notably, this represents one of the genuinely strong cases directly demonstrating physical PANoptosome assembly in a diabetic context: under hyperglycaemic conditions, enhanced glycolysis in retinal pigment epithelial (RPE) cells upregulates TXNIP expression via the epigenetic modification of histone H3 lysine 18 lactylation. TXNIP acts as a crucial bridge, driving the assembly of the NLRP3 inflammasome into a PANoptosome, ultimately triggering PANoptosis (99). Furthermore, intervention studies in DR models support the concurrent activation of these interconnected death mechanisms: Xu et al. found that intravitreal injection of Dickkopf-1 (DKK1) simultaneously suppressed markers of pyroptosis (cleaved GSDMD), apoptosis (caspase-3), and necroptosis (RIPK3) in the retinas of rats with DR, thereby exerting a protective effect. While this indicates broad PCD crosstalk, the physical assembly of a PANoptosome was not directly investigated in this specific model (100). Although the assembly of the TXNIP-NLRP3-mediated PANoptosome in DR RPE cells has been clearly demonstrated (99), the complete composition of this complex across different retinal cell types and its upstream regulatory networks remains to be further elucidated (**Figure 3**).

Targeting these integrated PCD networks represents a potential therapeutic strategy for intervening in DR. Directly targeting upstream metabolic pathways is a promising new direction: studies have shown that using the glycolysis inhibitor 2-deoxy-D-glucose or knocking down TXNIP *in vivo* and *in vitro* via adeno-associated virus-mediated shTXNIP can effectively block the glucose-driven ‘glycolysis-histone lactylation’ positive feedback loop, thereby inhibiting the activation of the NLRP3 inflammasome and the assembly of the PANoptosome, ultimately significantly reducing PANoptosis in retinal pigment epithelial cells (99). Furthermore, utilising factors with synergistic inhibitory functions is a practical strategy for achieving combined therapeutic effects. DKK1 serves as a prime example; when administered via intravitreal injection, it not only simultaneously inhibits markers of pyroptosis, apoptosis, and necroptosis in the DR retina, but also reduces pathological neovascularisation by modulating the Wnt/β-catenin pathway, thereby providing synergistic protection to the neurovascular unit (100). Collectively, this evidence constitutes a network of targets for potential therapeutic strategies against these interconnected PCD pathways in DR, laying a solid foundation for the development of effective combination therapies (**Table 1**).

In summary, given the limited efficacy of single-target inhibition in DR, targeting the integrated PCD network driven by hyperglycaemia and the glycolysis-histone lactylation axis offers superior potential; upstream metabolic interventions, sensors (e.g., NLRP3), and synergistic factors (e.g., DKK1) present promising therapeutic strategies, although the precise assembly of the retinal PANoptosome awaits definitive validation.

### 5.3 Diabetic neuropathy

DN is one of the most common and debilitating chronic complications of diabetes, characterised by progressive peripheral nerve damage induced by chronic hyperglycaemia and metabolic disturbances (101). According to current clinical evidence, at least 50% of people with diabetes worldwide will eventually develop DN, with a significant proportion suffering from severe symptoms such as chronic pain, sensory loss, and foot ulcers, which significantly increase the risk of amputation and lead to a severe decline in quality of life (101). Neuronal cell death is a key pathological factor mediating the progression of DN (102). There is substantial evidence that various forms of PCD, including pyroptosis, apoptosis, and necroptosis, are implicated in the pathogenesis of DN. For example, in a model of diabetic enteropathy, significant activation of necroptosis mediated by the RIPK3 and MLKL signalling pathways has been observed (103). A hyperglycaemic environment can also induce pyroptosis in Schwann cells, characterised by NLRP3 inflammasome activation, caspase-1 cleavage, and GSDMD cleavage (104). Concurrently, the apoptotic process in Schwann cells plays a significant role in diabetic peripheral neuropathy, a mechanism involving the regulation of complex signalling pathways (105).

Evidence suggests that concurrent activation of multiple PCD pathways is involved in the pathological process of DN. Innate immunity and metabolic stress play a central role in disease progression. The NLRP3 inflammasome, as a key innate immune sensor, acts as a crucial bridge linking the hyperglycaemic environment to nerve damage. In studies on DN, research by Wang et al. confirmed that hyperglycaemic treatment induces the simultaneous activation of pyroptosis pathways, represented by NLRP3/ASC/IL-1β, and apoptosis pathways, represented by caspase-3/9-Poly (ADP-ribose) polymerase in Schwann cells (106). Notably, this study further revealed that the removal of ROS simultaneously inhibits both pathways, suggesting that OS may be an upstream common mechanism triggering the parallel activation of apoptosis and pyroptosis in a high-glucose environment (106). This finding not only highlights the complexity of cell death in diabetic complications but also provides a crucial theoretical basis for exploring whether a PANoptosome exists in DN (106). Other core components of the PANoptosome also play key roles in driving nerve damage. Among these, necroptosis mediated by the RIPK3-MLKL pathway is a central mechanism leading to myelin damage. Research by Guo et al. demonstrated that MLKL is specifically activated in the myelin of Schwann cells in diabetic mouse models and patient nerve samples, and that its functional loss effectively prevents myelin degradation (107). Belavgeni et al. further characterised this process as ‘Schwann cell necroptosis’, establishing the pathway’s role in the disease (108). Furthermore, ERS serves as another crucial link between metabolic dysregulation and PANoptosis-related networks. Wang et al. found, in a diabetic encephalopathy model, that high-glucose-induced insulin resistance leads to impaired nuclear translocation of spliced X-box binding protein 1 (sXBP1). This event simultaneously relieves the inhibition on two downstream death pathways. On the one hand, sXBP1 deficiency results in abnormal activation of the activating transcription factor 6/CHOP axis, exacerbating ERS-mediated apoptotic signals. On the other hand, the NLRP3 inflammasome–caspase-1–GSDMD pathway is activated, triggering pyroptosis. Notably, the direct interaction between CHOP and p53 within the nucleus may constitute an integration node for apoptotic and pyroptotic signals, enabling both forms of cell death to occur simultaneously in DN, thereby exhibiting the characteristics of parallel PCD activation, although PANoptosome assembly was not explicitly visualised. Activation of sXBP1 can simultaneously reverse both of these death pathways, suggesting its pivotal role as an upstream regulatory hub of these interconnected PCD pathways (109). Crucially, research has revealed that specific metabolic stress molecules can act as upstream hubs, simultaneously initiating multiple cell death pathways. For example, upon upregulation in a hyperglycaemic environment, TXNIP can both induce mitochondrial apoptosis by binding to mitochondrial thioredoxin 2 and activate NLRP3 inflammasome-mediated pyroptosis, jointly leading to dorsal root ganglion damage (110). This strongly confirms that an integrated cell death network triggered by common upstream signals has a solid molecular basis in DN (**Figure 3**).

Targeting key components of these interconnected cell death networks is an effective strategy for alleviating DN. Direct inhibition of the core effector molecules of the PANoptosome components has been shown to mitigate nerve damage. For example, specific inhibition of MLKL function prevents demyelination mediated by Schwann cell necroptosis, thereby improving nerve conduction function (107, 108). Notably, targeting RIPK3 through pharmacological intervention has also demonstrated significant therapeutic potential. Cheng et al. found that in a model of diabetic enteropathy, the serotonin 4 receptor agonist RS67333 effectively inhibited RIPK3 activation, thereby reducing neuronal loss and intestinal dysfunction. Given RIPK3’s central role in PCD cross-regulation, this study not only confirms the benefits of inhibiting necroptosis but also suggests that targeting RIPK3 may represent a potential strategy for exerting neuroprotective effects by blocking these convergent death signals (103). Notably, targeting upstream regulatory hubs may enable more fundamental intervention in co-occurring PCD pathways. For instance, salvianolic acid B demonstrates therapeutic potential by scavenging ROS and simultaneously inhibiting hyperglycaemia-induced pyroptosis (NLRP3/caspase-1) and apoptosis (caspase-3) in Schwann cells, thereby targeting upstream OS to synergistically block multiple cell death pathways (106). Similarly, ERS is a key component in the pathogenesis of diabetic encephalopathy, and its upstream regulatory molecule, sXBP1, represents a more promising therapeutic target. Wang et al. demonstrated that isorhynchophylline can restore sXBP1 nuclear translocation, thereby improving insulin signalling while simultaneously inhibiting activating transcription factor 6/CHOP-mediated ERS and the consequent NLRP3-dependent pyroptosis and apoptosis, thus exerting multiple neuroprotective effects in diabetic encephalopathy (109). Furthermore, synergistic intervention with neurotrophic factors (such as nerve growth factor and basic fibroblast growth factor) can significantly reduce Schwann cell apoptosis by inhibiting ERS, thereby promoting nerve repair (111). Collectively, these studies indicate that intervention strategies targeting upstream nodes, such as ERS and ROS, can suppress multiple cell death programmes within this integrated network by modulating broader signalling networks, thereby offering new insights into the treatment of DN (**Table 1**).

In summary, targeting the integrated PCD network driven by chronic hyperglycaemic metabolic dysfunction offers synergistic protection in DN that surpasses single-pathway inhibition; consequently, combined interventions employing multi-target agents like Salvianolic acid B, or targeting upstream hubs (ERS, OS) and core executors (MLKL, RIPK3), represent fundamentally broader and highly promising therapeutic strategies.

### 5.4 Diabetic cardiomyopathy

DCM is a major cardiac complication of diabetes, characterised by structural and functional abnormalities of the myocardium in the absence of coronary artery disease or hypertension, which can ultimately lead to heart failure (72, 112). It has been demonstrated that all three programmes of cell death—pyroptosis, apoptosis, and necroptosis—are jointly involved in the pathogenesis of DCM (51). These PCD pathways do not exist in isolation within the pathological context of DCM, but rather form a complex network of interactions. Several studies have shown that inhibiting a single type of PCD in DCM often fails to prevent overall cardiomyocyte loss and disease progression. For example, whilst inhibiting the TLR4 signalling pathway using TAK-242 alleviates pyroptosis, it does not fully reverse myocardial damage (113). Similarly, the application of the necroptosis inhibitor Necrostatin-1, whilst suppressing RIPK1 activity and improving fibrosis, may activate other forms of cell death (114). Collectively, these findings reveal that within the complex pathological network of DCM, there is significant compensation and crosstalk among PCD pathways, imposing fundamental limitations on interventions targeting a single pathway. This has prompted researchers to shift their focus towards higher-order, integrative cell death networks, such as PANoptosis.

Recent studies have provided compelling evidence for the concurrent activation of multiple PCD pathways in DCM, suggesting a potential role for PANoptosis-like networks. Guan et al. found that in high-glucose cardiomyocyte models and db/db diabetic mouse models, the circular RNA circular RNA OGDH (circOGDH activates multiple PCD pathways in DCM by regulating the High mobility group box 1-RIPK3 axis, thereby promoting diabetic cardiomyocyte death; inhibition of circOGDH significantly reduces the expression of these integrated cell death markers (115). Li et al. and Wang et al. confirmed through *in vivo* and *in vitro* functional experiments that AIM2, a key innate immune sensor, is significantly upregulated in DCM (116, 117). Inhibition of AIM2 effectively reduces hyperglycaemia-induced cardiomyocyte death and cardiac fibrosis, thereby improving cardiac function. Furthermore, the deacetylase sirtuin 3 (SIRT3), a key regulator of mitochondrial function and OS, shows dysregulated expression closely associated with DCM. Song et al. utilised SIRT3 knockout mouse models and high-glucose-treated neonatal rat cardiomyocytes to demonstrate that SIRT3 deficiency exacerbates hyperglycaemia-induced myocardial mitochondrial damage and ROS accumulation, whilst promoting the expression of proteins associated with necroptosis (as evidenced by elevated levels of RIPK1 and RIPK3) and apoptosis (with increased cleaved caspase-3 expression), and simultaneously activating the NLRP3 inflammasome (accompanied by increased levels of caspase-1 p20 and IL-1β), ultimately exacerbating DCM. This study provides experimental evidence for understanding the role of SIRT3 in regulating multiple modes of cell death in DCM. However, as no significant changes in MLKL were observed, the necroptosis arm is undercut in this model. Therefore, rather than a canonical PANoptosis event, this study robustly demonstrates profound co-occurring crosstalk among PCD pathways, highlighting the mechanistic complexity in DCM without definitively proving PANoptosome assembly (**Figure 3**) (118).

Targeting these integrated PCD networks has emerged as a potential therapeutic strategy for mitigating DCM. Several studies have confirmed that AIM2 and NLRP3, key sensors often implicated in PANoptosome assembly, are significantly up-regulated in DCM; inhibiting these two molecules effectively reduces hyperglycaemia-induced cardiomyocyte death and cardiac fibrosis, thereby improving cardiac function (116, 117). Further research indicates that targeting upstream regulatory genes can achieve simultaneous inhibition of pyroptosis, apoptosis, and necroptosis, thereby more effectively delaying the progression of DCM. For example, knockdown of circOGDH significantly improves cardiac function and reduces myocardial fibrosis *in vitro*, whilst also suppressing the expression of multiple PCD-associated proteins, suggesting that it may serve as a nucleic acid therapeutic target for DCM (115). Wang et al. utilised cluster of differentiation 38 (CD38) global knockout mice and cardiomyocytes exposed to high glucose and palmitic acid to demonstrate that CD38 deficiency, by activating the NAD^+^/SIRT3/forkhead box O3a signalling pathway, significantly inhibits pyroptosis and apoptosis in cardiomyocytes, whilst improving glucose metabolism and cardiac function (119). Furthermore, Han et al. transfected high glucose-induced H9c2 cells with epithelial membrane protein 1 siRNA. They found that epithelial membrane protein 1 knockdown, by inhibiting the RAS/RAF/MAPK pathway, reduced NLRP3 inflammasome activation and the expression of its downstream pyroptosis markers (GSDMD, cleaved caspase-1, IL-1β) and apoptosis markers (cleaved caspase-3), whilst simultaneously alleviating mitochondrial damage and OS (120). In summary, targeting these interconnected PCD networks and its upstream regulatory molecules shows great promise for treating DCM; however, further exploration of its clinical translation potential is required (**Table 1**).

In summary, targeting integrated PCD networks offers a superior therapeutic strategy for DCM compared to single-pathway inhibition, supported by the synergistic regulation of key molecules like circOGDH, AIM2, and SIRT3; nevertheless, the lack of direct evidence for functional PANoptosome assembly in cardiomyocytes remains a core translational bottleneck.

### 5.5 Diabetic foot ulcer

DFU is a severe complication of diabetes, characterised by chronic wounds, a high inflammatory response, and a high risk of amputation, with a rising global prevalence (1, 121). While pyroptosis, apoptosis and necroptosis are known to contribute to DFU pathology (122–124), it is crucial to foreground that there are currently no reported studies directly demonstrating PANoptosome assembly in clinical DFU tissue. Consequently, the evidence presented in this section rests primarily on highly relevant surrogate models (such as limb ischaemia, ischaemic flaps, and diabetic full-thickness wounds). Extrapolating from these models, there is strong theoretical and indirect evidence suggesting that interconnected PCD networks play a pivotal role in the pathological progression of DFU. The refractory nature of DFU healing is inextricably linked to core pathological factors such as lower limb ischaemia, neuropathy, and persistent infection, which collectively constitute a complex inflammatory and metabolic microenvironment (125). In recent years, numerous studies using surrogate models have elucidated the mechanisms underlying PANoptosis activation in diabetes-related tissue damage from various perspectives, providing important insights into the pathogenesis of DFU.

Ischaemia is one of the key drivers of DFU, and the combined effects of hyperglycaemia and hypoxia can synergistically induce PANoptosis. Wang et al. directly observed the assembly of the PANoptosome in vascular endothelial cells for the first time in a mouse model of diabetic lower limb ischaemia. Importantly, representing one of the genuinely strong cases in the diabetic context, this study provided direct evidence by explicitly visualising PANoptosome assembly. It confirmed that hyperglycaemia combined with ischaemia upregulates cytochrome P450 2E1 expression, triggering a burst of mitochondrial ROS and impairing mitophagy, thereby activating PANoptosis, leading to endothelial dysfunction and impaired angiogenesis. Treatment with Pseudostellaria heterophylla polysaccharides was shown to block endothelial cell PANoptosis by inhibiting cytochrome P450 2E1 expression and promoting angiogenesis (126). Further studies indicate that ischaemia-reperfusion injury itself can also influence PCD networks by regulating autophagy. Yu et al. found in a diabetic mouse ischaemic flap model that ischaemia activates the miR-107-3p/Wnt3a/mTOR/TFEB (transcription factor EB) signalling axis, inhibits autophagic flux, and promotes the upregulation of key PCD-associated components (such as RIPK3 and NLRP3). Conversely, treatment with the caloric restriction mimetic 3,4-dimethoxychalcone effectively suppressed these concurrent death signals and improved tissue survival by downregulating miR-107-3p, activating the Wnt3a/β-catenin pathway, and thereby restoring autophagic flux in ischaemic flaps (127). These findings suggest that the reciprocal regulation between autophagy and PCD crosstalk may represent a critical pathological mechanism within the ischaemic microenvironment of DFU (**Figure 3**).

In addition to ischaemic factors, local metabolic disturbances and persistent inflammation at the DFU wound site also drive the occurrence of integrated cell death. Xue et al. demonstrated in a model of extensive skin defects that a core-shell nanofibre scaffold loaded with bone marrow mesenchymal stem cell exosomes significantly downregulated the expression of multiple PCD-related genes, including NLRP3, CARD10, and ZBP1, and suppressed the release of pro-inflammatory cytokines such as IL-1β and IL-6, thereby accelerating wound healing (128). This study elucidates the role of the metabolic homeostasis–PCD axis in skin regeneration, suggesting that dysregulated glucose and lipid metabolism in DFU may exacerbate cell death via similar mechanisms. More direct evidence comes from studies focusing on diabetic wounds themselves. Wang et al. found that TAK1 expression was significantly upregulated in a diabetic full-thickness skin defect model in db/db mice under hyperglycaemic conditions. By directly binding to p65, it activated the NF-κB signalling pathway, promoting ZBP1/ASC/AIM2-mediated PANoptosome assembly, inducing PANoptosis in keratinocytes and fibroblasts, whilst simultaneously exacerbating local inflammatory responses and microvascular damage. Local knockdown of TAK1 effectively reversed these changes, confirming that the TAK1-NF-κB-PANoptosis axis is a core mechanism underlying impaired diabetic wound healing (**Table 1**) (129).

In summary, although direct clinical evidence remains lacking, surrogate models suggest that extensive PCD crosstalk and ischaemia-induced PANoptosis are driven by complex networks—including the cytochrome P450 2E1-ROS, miR-107-3p/autophagy, metabolic reprogramming, and TAK1-NF-κB axes—making the targeted inhibition of these nodes to prevent massive cell death in endothelial cells, keratinocytes, and fibroblasts a highly promising strategy for DFU healing.

## 6 Conclusions and prospects

PANoptosis, as an inflammatory form of cell death that integrates pyroptosis, apoptosis, and necroptosis, is reshaping our understanding of the synergistic pathogenic mechanisms underlying metabolic stress and innate immunity. This review systematically summarises the key role of PANoptosis in the onset and progression of diabetes and its complications, emphasising that it not only reveals the coexistence of these three cell death pathways in the diabetic state, but also provides a unified molecular explanation for their extensive interactions and compensatory activation. Building on this, the paper summarises the current state of research, analyses existing knowledge gaps, and outlines future research directions and therapeutic strategies.

Beyond the shared molecular machinery, a crucial unanswered question with direct clinical implications is whether PANoptosis patterns differ between type 1 and type 2 diabetes mellitus. Although PANoptosis is implicated in both type 1 and type 2 diabetes mellitus, no study has directly compared them, with current evidence mainly derived from unstratified complication studies (19). Given their distinct pathophysiologies, we propose that type 1 diabetes mellitus PANoptosis is primarily immune-driven via the IFN-γ/TNF-α-mediated Janus kinase (JAK)/STAT1–IRF1 axis (5, 19) following infiltration by autoreactive T cells and M1 macrophages, whereas type 2 diabetes mellitus PANoptosis is mainly metabolically triggered by hyperglycaemia (which induces advanced glycation end products and mitochondrial ROS to activate AIM2/ZBP1) and by free fatty acids (which engage TLR4/NLRP3) (19) across β-cells, adipocytes, and endothelial cells. This divergence is reinforced by the type 2 diabetes mellitus-specific DAMP islet amyloid polypeptide (19, 130) and distinct non-coding RNA regulatory networks (131). Therefore, future subtype-stratified studies are urgently needed to validate these hypotheses and to inform precision therapeutic strategies.

The PANoptosome, as the central prerequisite for PANoptosis, can coordinate the activation of multiple cell death effectors and has found strong support in the context of diabetes. Current research focuses on several key themes: firstly, metabolic stress is the primary trigger. Chronic hyperglycaemia, lipotoxicity, ERS, and mitochondrial dysfunction are potent inducers of PANoptosis. These stimuli converge on innate immune sensors (such as NLRP3, AIM2, and ZBP1), which act as hubs integrating metabolic danger signals with the cell death machinery. Recently elucidated pathways, such as the ‘glycolysis–histone lactylation–TXNIP’ axis in DR and the ERS-CHOP-TXNIP axis in DKD, illustrate how metabolic disturbances are linked to PANoptosome activation at the transcriptional and epigenetic levels. Second, the diversity of PANoptosomes in diabetic complications: six major PANoptosome types have been identified to date. Among them, the NLRP3- and NLRC5-PANoptosomes represent the latest advances in the field. The RIPK1-PANoptosome, acting as a hub for the integration of inflammatory signals, plays a central role in multiple complications; the AIM2-PANoptosome, which senses mitochondrial DNA released upon mitochondrial damage, is well-documented in DKD and DCM; the ZBP1-PANoptosome has been shown to participate in PANoptosome assembly in surrogate models mimicking the DFU microenvironment; and the NLRP12-PANoptosome drives organ damage in haemolytic models, although its significance in diabetes remains to be explored. It is worth noting that NLRP3 exhibits a crucial dual role: while it frequently functions as an integrated downstream component recruited into other PANoptosomes (such as those nucleated by ZBP1 or AIM2), recent evidence confirms it can also independently nucleate the NLRP3-PANoptosome under specific metabolic stressors. As the most extensively studied sensor, it has been implicated in all five major complications and has been directly confirmed to participate in PANoptosome assembly in DR. Third, the necessity of multi-target inhibition: A consistent finding across all complications is that inhibiting a single PCD pathway yields limited efficacy. Compensatory activation of alternative death pathways often undermines single-therapy strategies. This mechanistic redundancy underscores the therapeutic rationale of targeting upstream PANoptosome assembly (e.g., via RIPK1, ASC, or specific sensors) or employing multi-targeted drugs (e.g., DKK1, Salvianolic acid B, Panax notoginseng polysaccharides). Such approaches, capable of simultaneously inhibiting multiple death effector pathways, have demonstrated superior efficacy in preclinical models of DKD, DR, and surrogate diabetic wounds. Finally, PANoptosis is not merely a mode of cell loss; it is a highly inflammatory event. DAMPs and pro-inflammatory cytokines (IL-1β, IL-18) released during the lysis of dying cells fuel a vicious cycle of chronic low-grade inflammation, immune cell recruitment, and further tissue damage. This links PANoptosis directly to the hallmark inflammatory pathogenesis of diabetic complications, affecting the vascular system, neurons, and parenchymal cells.

Although the role of PANoptosis in diabetes is becoming increasingly evident, several key issues must be resolved to bridge the gap between mechanistic understanding and clinical translation. Firstly, direct observation and comprehensive analysis of PANoptosome components in human diabetic tissues or relevant human cell models remain scarce. Future research should employ techniques such as proximity analysis, immunoprecipitation coupled with mass spectrometry, and advanced microscopy to confirm the physical assembly of PANoptosomes—comprising NLRP3, caspase-8, RIPK3, and others—in patient-derived samples. Secondly, the manifestation of PANoptosis may vary depending on cell type (e.g., endothelial cells, podocytes, cardiomyocytes, Schwann cells, β-cells) and disease stage. Single-cell and spatial transcriptomics/proteomics in diabetic tissues will be invaluable for mapping the cell-specific expression profiles of PANoptosis components and identifying the most vulnerable cell populations. Understanding the chronological sequence of PANoptosome assembly and activation during disease progression is also crucial for determining the optimal therapeutic window. Thirdly, current interventions typically rely on inhibitors of single PCD pathways (such as Necrostatin-1 and caspase inhibitors). There is an urgent need to develop specific drugs that can effectively disrupt PANoptosome assembly without affecting the homeostatic functions of its components; however, such agents remain scarce at present. In the future, structure-based drug design and high-throughput screening should be employed to identify small molecules or biologics capable of specifically interfering with key protein-protein interactions within the PANoptosome (such as the recruitment of ZBP1-RIPK3 or ASC-caspase 1). Concurrently, research could focus on rational combination therapies that simultaneously target upstream metabolic triggers (such as SGLT2 inhibitors and GLP-1 receptor agonists) and downstream PANoptosis execution pathways. Consideration should also be given to patient-specific factors (genetic and microbiome-related) that may influence susceptibility to PANoptosis.

In summary, PANoptosis represents a unified and targetable mechanism driving inflammatory cell loss in diabetic complications. Shifting the research focus from mechanistic discovery in animal models to translational validation in human disease is crucial to developing a new generation of therapies to halt the progression of these debilitating conditions.
